# Automatic analysis of summary statements in virtual patients - a pilot study evaluating a machine learning approach

**DOI:** 10.1186/s12909-020-02297-w

**Published:** 2020-10-16

**Authors:** Inga Hege, Isabel Kiesewetter, Martin Adler

**Affiliations:** 1 Medical Education Sciences, University of Augsburg, Augsburg, Germany; 2 Institute for Medical Education, Klinikum der Ludwig-Maximilians-Universität München, Pettenkoferstr. 8a, 80336 Munich, Germany; 3 Department of Anaesthesiology, Klinikum der Ludwig-Maximilians-Universität München, Munich, Germany; 4 Instruct gGmbH, Kapuzinerstr.5, 80337 Munich, Germany

**Keywords:** Clinical reasoning, Summary statement, Virtual patients, Natural language processing, Machine learning

## Abstract

**Background:**

The ability to compose a concise summary statement about a patient is a good indicator for the clinical reasoning abilities of healthcare students. To assess such summary statements manually a rubric based on five categories - use of semantic qualifiers, narrowing, transformation, accuracy, and global rating has been published. Our aim was to explore whether computer-based methods can be applied to automatically assess summary statements composed by learners in virtual patient scenarios based on the available rubric in real-time to serve as a basis for immediate feedback to learners.

**Methods:**

We randomly selected 125 summary statements in German and English composed by learners in five different virtual patient scenarios. Then we manually rated these statements based on the rubric plus an additional category for the use of the virtual patients’ name. We implemented a natural language processing approach in combination with our own algorithm to automatically assess 125 randomly selected summary statements and compared the results of the manual and automatic rating in each category.

**Results:**

We found a moderate agreement of the manual and automatic rating in most of the categories. However, some further analysis and development is needed, especially for a more reliable assessment of the factual accuracy and the identification of patient names in the German statements.

**Conclusions:**

Despite some areas of improvement we believe that our results justify a careful display of the computer-calculated assessment scores as feedback to the learners. It will be important to emphasize that the rating is an approximation and give learners the possibility to complain about supposedly incorrect assessments, which will also help us to further improve the rating algorithms.

## Background

Clinical reasoning is a complex set of skills healthcare students have to acquire during their education. Apart from face-to-face teaching scenarios, such as bedside teaching, clinical reasoning can be trained with web-based virtual patients (VPs) [1]. VPs are scalable, allow for deliberate practice, and provide a safe environment in which neither students nor patients are harmed.

CASUS [2] is a virtual patient software, that supports clinical reasoning training in multiple ways - with a variety of question types, a specific clinical reasoning tool [3], and the composition of a summary statement by the learners.

Summary statements are short presentations of a patient of usually one to three sentences length. The ability to present a patient in such a concise way is a good indicator for clinical reasoning skills, because the student has to summarize and synthesize a patient’s information [4]. In CASUS, learners currently get feedback in form of a static expert statement after having submitted their own statement, but the statements are not yet assessed in an automatic way, thus, no dynamic and individual feedback is provided.

Smith et al. have developed a rubric to assess the quality of such summary statements and provide structured feedback to learners [5]. Their rubric includes five components - factual accuracy, appropriate narrowing of the differential diagnosis, transformation of information, use of semantic qualifiers (SQ), and a global rating. Each component can be rated on a two- or three-point scale. With this detailed assessment considering different aspects the rubric can help learners to monitor and assess their progress. However, this approach is based on human raters; for an implementation for real-time rating and feedback in VPs, the summary statements have to be analyzed automatically.

In the recent years natural language processing (NLP) and machine learning (ML) tools became more accessible as services and have also been applied in medical education [6]. Such techniques aim to enable computers to parse and interpret spoken or written human language as humans would do [6].; for example, Denny at al. describe the use of NLP to identify competencies from students’ clinical notes [7] and Spickard et al. extracted and cataloged concepts from students’ clinical notes to track their progress [8].

The aim of our project was to combine the rubric by Smith et al. with NLP approaches to test whether an automatic real-time assessment of summary statements can serve as a basis for providing structured qualitative feedback to learners without the need of manually training such a system on a VP-based level.

## Implementation

From January 2017 to July 2019 100 virtual patients in German and English were provided in two open-access courses in CASUS to healthcare students world-wide as a voluntary and self-directed training opportunity [2]. Each expert-reviewed VP included a clinical reasoning tool that was developed to specifically support the clinical reasoning skills acquisition with a concept mapping approach [3]. Additionally, in each VP learners were prompted to compose a short statement summarizing the patients history; a brief introductory video explained the purpose and main components of such a statement [9]. Feedback was provided in form of an exemplary summary statement composed by the VP author. Overall, during this period of data collection, learners created 1505 summary statements in German and English.

For the purpose of this project we selected five VPs covering a broad range of key symptoms, such as fever, abdominal pain, or dyspnea with acute or chronic courses of disease and covering different final diagnoses, such as asthma, colitis ulcerative, or pneumonia. From these five virtual patients we randomly selected 125 summary statements in both languages and collected them in an excel file. Two healthcare professionals (IK, IH) independently rated the 125 statements based on the assessment rubric published by Smith et al. (Table 1). Additionally, to emphasize a patient-centered approach, we included a new category to assess whether the patient was addressed with his or her name in the statements. After studying and discussing the assessment rubric (Table 1) the two healthcare professionals independently rated 25 statements followed by a discussion about any divergent codings. After reaching consensus in all categories the remaining 100 statements were coded. Disagreements among the raters were solved in a personal discussion and consensus was reached in all cases.

**Table 1:** Rating rubric suggested by Smith et al. (0 = None, 1 = Some, 2 = Appropriate) [5] and additional category “patient name”

Category	Scoring	Description
Use of semantic qualifiers	0, 1, or 2	Use of qualitative terms (e.g. “acute”, “unilateral”, “severe”)
Appropriate narrowing of differential diagnosis	0, 1, or 2	Including key features to narrow the differential diagnosis
Transformation of information	0, 1, or 2	Use of medical terminology (e.g. “Fever” instead of Temperature: 39.4 °C”
Factual accuracy	0 (No), 1(Yes)	Only accurate information included
Patient name	0 (No), 1 (Yes)	The (virtual) patient is addressed by name and not called “the patient”.
Global rating	0, 1, or 2	Overall rating

Based on a focused internet research we evaluated potential NLP tools and software solutions, that could support the analysis of summary statements by creating a semantic structure of the written texts. We decided to try the python framework spaCy [10] because it is

- applicable for summary statements in English, German, and potentially other languages
- potentially applicable for real-time assessment via an API
- open-source.

For optimal results, we followed a two-step approach combining available metadata of the VP for each category and the controlled vocabulary thesaurus MeSH ((Medical Subject Headings) and an analysis with spaCy.

First, we used the spaCy tree to assess the five components of the rubric and the additional patient category (see Table 2).

**Table 2:** Computer-based calculation of the scores in the six categories

Category	Method	Score formula
Use of semantic qualifiers (SQ)	Identification of semantic qualifiers in the statements based on the list provided by Connell et al. [11] and application of rules to compare results, occurrences and the semantic context with the NLP tree.	< 2 SQ: Score = 0 > = 2 and < =4 SQ: Score = 1 > 4 SQ: Score = 2
Appropriate narrowing of differential diagnosis	Identification of findings, differential diagnoses, and anatomical terms based on an adapted MeSH thesaurus and comparison of the result with analysis of the expert statement and VP metadata.	(found terms of expert - terms of learner matching with expert -) / found terms of expert: > 0.75: Score = 0 <= 0.75 and > = 0.25: Score = 1 < 0.25: Score = 2
Transformation of information	Identification of transformed terms and non-transformed terms based on a list of SI units and the MeSH thesaurus and comparison with transformed terms by expert and overall length of the statement.	(transformed terms - non-transformed terms /2)/ (transformed terms of expert + text length factor) < 0.16: Score = 0 > = 0.16 and < = 0.7: Score = 1 > 0.7: Score = 2
Factual accuracy	Identification of contradicting use of SQ in the learner and expert statement	contradicting information found: score = 0, else score = 1.
Patient name used	Identification of a person token in the NLP tree	person identified: score = 1, else score = 0.
Global rating	Sum of the five categories	Sum <=2: Score = 0 Sum > 2 and < =5: Score = 1 Sum > 5: Score = 2

Second, we created with spaCy a tree of entities, sentences, and tokens of the summary statements.

For both steps we applied general rules and no VP-specific algorithms to guarantee the applicability of our approach for a broad variety of VPs.

For real-time feedback the time needed to calculate the rating is an important factor, thus, we optimized the algorithm in terms if performance and recorded the time needed for the longest summary statement.

For comparing the manual and the automatic rating we calculated Cohen’s kappa using SPSS version 26, with values of 0.01 to 0.20 considered none/slight, 0.21 to 0.40 fair, 0.41 to 0.60 moderate, 0.61 to 0.80 substantial, and 0.81 to 1.00 almost perfect agreement.

We received ethical approval from the Ethical Committee of the University of Munich for the anonymous collection and analysis of summary statements.

## Results

The comparison of manual and computer-based rating in the six categories is shown in Table 3. The detailed results for 50 exemplary summary statement can be found in Additional file 1.

**Table 3:** Comparison of manual (columns) and automatic (rows) rating of summary statements in the six categories and Cohen’s kappa as measure of agreement between the manual and the automatic rating

Category	Automatic trating	Manual rating			Congruent rating
		0	1	2	
Semantic qualifiers	**0**	39	15	0	75.2%, κ = .557
	**1**	5	51	9	
	**2**	0	2	4	
Appropriate narrowing	**0**	21	9	1	81.6%, κ = .458
	**1**	8	68	13	
	**2**	0	2	3	
Transformation	**0**	47	14	1	69.6%, κ = .484
	**1**	11	35	5	
	**2**	0	6	5	
Factual accuracy	**0**	5	2	–	93.6%, κ = .366
	**1**	12	106	–	
Patient name	**0**	78	10	–	90.4, κ = .783
	**1**	2	35	–	
Global rating	**0**	24	4	0	80.0%, κ = .582
	**1**	8	72	5	
	**2**	0	8	4	

Overall, Table 3 shows a substantial agreement (κ > = .61) between the manual and the automatic rating in the category “patient name”, a fair agreement for the category “factual accuracy” and moderate agreement (κ > =.41) for all other categories. Complete mismatches with a rating distance of 2 can be seen in two categories (appropriate narrowing and transformation) each showing one manual rating with a 2 and an automatic rating with 0.

When looking into the results of the analysis of German and English summary statements, we detected some issues in the “patient name” category. The NLP identified all 35 persons in the English statements, with two false hits, but for German statements none of the 10 patient names were identified.

The following shows an example of a summary statement for a VP with tuberculosis: “67 year old patient, presents with a cough that lasted 3 months. Has a smoking history. Has experienced weight loss and loss of appetite. Green sputum. Earlier diagnosed with hypertension, treated in China.”

**Fig. 1:**
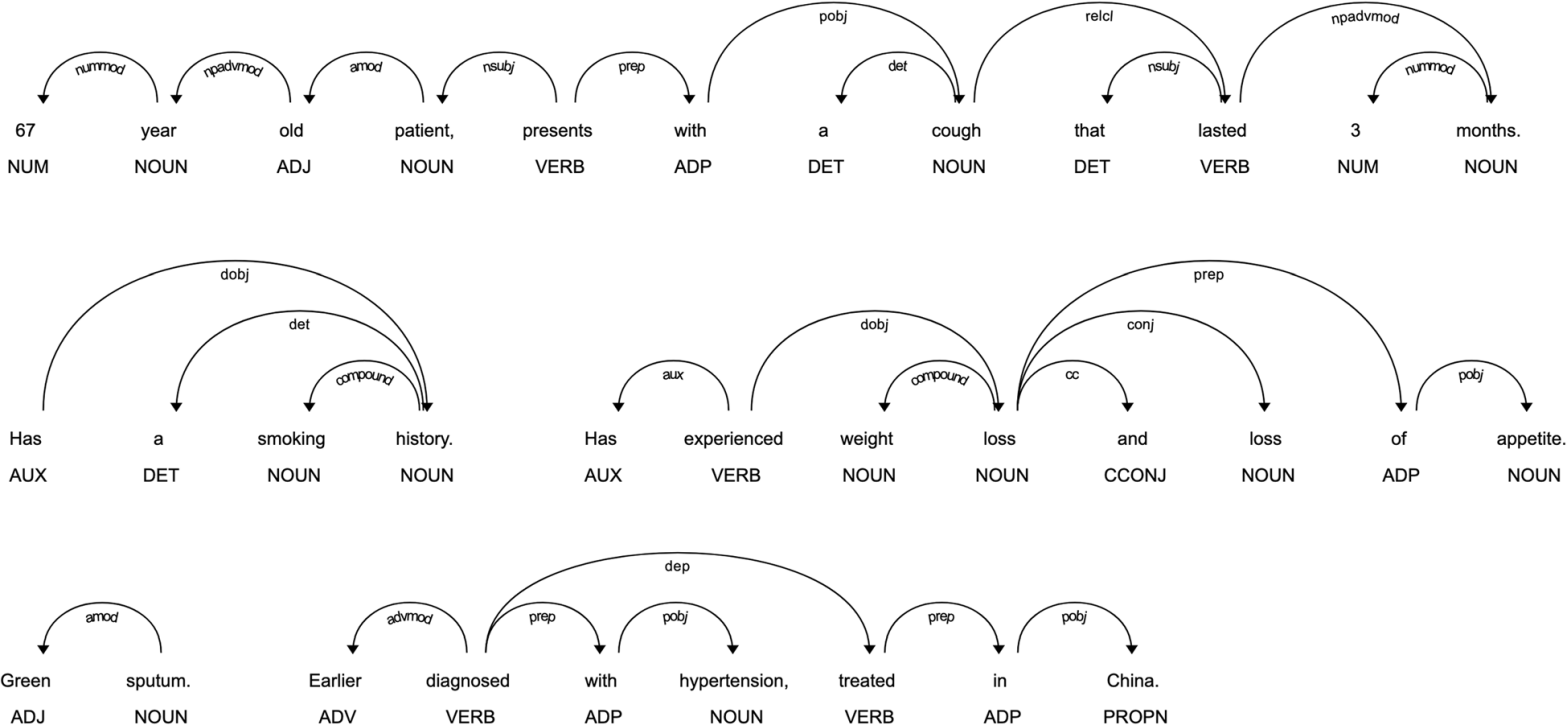
NLP tree of an exemplary summary statement indicating the type of entity, such as noun, verb, or adjective and the type of dependencies between entities. For example, “3” is a numeric modifier (nummod) for “months”. The list of annotations can be found at https://spacy.io/api/annotation

The NLP tree of this statement is shown in Fig. 1.

Our algorithm was then able to identify and classify the following terms:

“67 year old (date) patient, presents with a cough (finding) that lasted 3 months (duration). Has a smoking (finding) history. Has experienced weight loss (finding) and loss of appetite (finding). Green sputum (anatomical term). Earlier diagnosed with hypertension (diagnosis, hyper = SQ), treated in China (country).”

This leads to the following calculated scores:

1. SQ = 0 (1 SQ identified)
2. Appropriate narrowing = 1 (3 matching terms with expert statement or VP metadata)
3. Transformation = 1 (2 terms indicating a transformation)
4. Accuracy = 1 (no incorrect information identified)
5. Patient name = 0 (no patient name identified)
6. Global rating = 1 (sum = 5)

The exemplary statement was similarly assessed by the rater, only the transformation was rated with 0 instead of 1.

The measured time for the summary statement analysis was on average 1.3 s, with a maximum of 5.8 s for the longest statement.

## Discussion

The aim of our project was to test whether an automatic rating of summary statements based on the rubric provided by Smith et al. can be used for providing real-time feedback to learners by applying general rules without having to train a system specifically for a VP. Overall, we believe that the results of our combined approach for the six components are promising showing a moderate agreement between the manual and automatic agreement for most of the categories and only very few complete mismatches with a rating distance of 2.

For some components, we identified difficulties in achieving more reliable results: The main challenge in the category “patient name” were German statements in which we could not identify names or persons at all due to the limitations of the NLP model. This could be solved by providing the name of the VP as metadata and compare it directly with the statement.

With only a slight agreement (κ = .366) especially the category Factual Accuracy requires further refinement. From our 125 randomly selected summary statements only 17 were rated as not accurate in the manual assessment and only five of these were then correctly identified with our algorithm. This low number and the great variety of potential errors in statements makes it difficult to achieve a more reliable detection of non-accurate statements. To further improve our algorithm to detect errors, we will have to specifically collect and analyze more non-accurate statements. Despite the importance of accuracy for the rating of a statement, it seems a difficult category to rate, for which also in the study by Smith et al. interrater reliability was lowest [5]. Their plan for improvement was the further development of the rubric from a binary to a multiple-option category. Such a specification might also help to further develop our algorithm to categorize and detect potential error sources.

In contrast to the rating rubric by Smith et al. we calculated a more specific ratio for all categories except patient name, factual accuracy, and global rating, which was then translated by thresholds into the 0,1,2 - rubric. In doing so, we lost some information, that could give learners a better and more accurate understanding on their performance.

The analysis of the summary statement is a complex task, requiring an average of 1.3 s per statement, with 58 of the longer statements requiring more than 1 sec, which is according to Nielsen the limit for an uninterrupted flow of thought [12]. Hence, displaying the analysis results as real-time feedback to the learners in their learning analytics dashboard will require a pre-calculation in the background guaranteeing an uninterrupted user experience.

### Limitations

For our project, we randomly selected 125 statements from five VPs, which is quite a low number compared to the overall number of summary statements already collected and the number of VPs available in the CASUS system. When selecting the VPs for the project our aim was to cover a broad spectrum of findings and differential diagnoses, but we cannot exclude that for specific VPs the algorithm might return less accurate ratings. More testing with a higher number of summary statements of the five VPs and additional VPs has to be implemented to further validate our results. Finally, we cannot exclude that due to a volunteer bias the summary statements are more homogenous than without such a bias. However, assuming that volunteer learners tend to be more motivated and engaged [13], but also having only a few statements with a global rating of 2 (see Table 3) we believe that it is unlikely that such a bias had an influence on our results. Unfortunately, we do not have similar studies to compare our results to,

## Conclusions

Overall, most of the categories show a moderate agreement between the manual and the automatic rating, which we think is a justifiable starting point for a careful feedback to the learners about their performance in summary statement composition as part of the learning analytics dashboard. However, we would refrain from displaying the absolute rubric scores (0,1, or 2), but the underlying ratio in each category. It will also be important to emphasize the possibility of false interpretations of the automatic rating and give learners the chance to provide feedback concerning the assessment of their statement. This feedback will also form an important step in further improving our algorithm.

Apart from analyzing summary statements, our approach might also be a first step for analyzing other texts composed by healthcare students, such as e-portfolio entries.

## Availability and requirements

Project name: Effective clinical reasoning in virtual patients

Project home page: https://github.com/clinReasonTool

Operating system(s): Platform independent

Programming language: Java, Python

Other requirements: none

License: e.g. MIT

Any restrictions to use by non-academics: none

## Supplementary information

**Additional file 1.** Exemplary 50 summary statements with manual and automatic rating. The appendix contains 50 analyzed English summary statements with the manual and automatic rating based on the rubric and the time of the automatic assessments.

## Data Availability

The dataset with the 125 summary statements in English and German including the results of the manual and automatic rating can be obtained from the authors, an exemplary dataset of 50 summary statements in English is included in this article as Additional file 1.

## Abbreviations

NLP: Natural language processing
SQ: Semantic qualifiers
VPs: Virtual patients
